# Associations of Musical Activities and Positive Affect With Fear of Childbirth: A Structural Equation Modeling Approach

**DOI:** 10.3389/fpubh.2022.906996

**Published:** 2022-06-17

**Authors:** Shidi Liu, Yi Jin, Hongmei Li, Tingting Zeng, Ge Zhou, Lili Yu, Yao Fan, Xun Lei

**Affiliations:** ^1^School of Public Health and Management, Chongqing Medical University, Chongqing, China; ^2^Research Center for Medicine and Social Development, Chongqing Medical University, Chongqing, China; ^3^Collaborative Innovation Center of Social Risks Governance in Health, Chongqing Medical University, Chongqing, China; ^4^Research Center for Public Health Security, Chongqing Medical University, Chongqing, China; ^5^Obstetrics and Gynecology Center, The Third Affiliated Hospital of Chongqing Medical University, Chongqing, China

**Keywords:** fear of childbirth, musical activities, positive affect, structural equation meddling, interventions

## Abstract

**Background:**

Fear of childbirth is a prevalent issue among women, with a wide range of interventions to dispel it. Here we explored a novel and beneficial intervention and one possible influence mechanism of it.

**Methods:**

The cross-sectional study recruited 1,053 pregnant women from one tertiary-grade A class hospital between March to August 2021. The questionnaire included demographic characteristics, a self-made musical activities questionnaire, the Positive affect subscale, and the Childbirth Attitudes Questionnaire. We parceled the eight musical activities into three items by item parceling methodology. The associations of musical activities and positive affect with fear of childbirth were evaluated by a structural equation modeling approach.

**Results:**

Our analyses demonstrated the effectiveness of musical activities, which was notably correlated with the increase in positive affect (β = 0.309, *P* < 0.01). On the contrary, positive affect predicted a decrease in fear of childbirth (β = −0.085, *P* = 0.019). Additionally, positive affect mediated the effect of musical activities on fear of childbirth (β = −0.026, *P* = 0.030). However, the direct effect of musical activities on fear of childbirth was not found (β = 0.029, *P* = 0.514).

**Conclusions:**

Relying on musical activities alone may not be adequate to alleviate the fear of childbirth, and positive affect played a pivotal role between musical activities and fear of childbirth. The results showed that musical activities would be an effective non-pharmaceutical way to alleviate the fear of childbirth and positive affect can not be ignorant in future childbirth fear reduction programs.

## Introduction

Childbirth, a life-changing event for a woman, puts her physical and mental health in danger. A variety of questionnaires have been used to assess childbirth fear. According to O'Connell et al. (1), the combined prevalence of severe fear of childbirth in Western and Asian countries was 14%. In Australian studies, the prevalence was 23%, in American studies, 11%, and in Asian studies, 25%, all of which were greater than the other studies and the average. Clearly, the number of pregnant women who are afraid of childbirth is considerably larger than the figures above. Despite the fact that modern obstetrics has reached unprecedented levels of sophistication, pregnant women's anxiety of childbirth appears to be on the rise. According to Räisänen et al. (2), the prevalence of fear of childbirth (FOC) in Finland increased from 1.1 to 3.6 percent in nulliparous women and from 1.5 to 7.8 percent in multiparous women between 1997 and 2010. Furthermore, when O'Connell et al. (1) looked at the prevalence of severe FOC across time, she found that it was lower in the earlier years (the 1980s, 1990s) but grew in more recent years. Consequently, the massive amount of FOC spreading among women sparks health concerns and prompts researchers from all walks of life to alleviate it.

Fear of childbirth is a prevalent feeling experienced by women before and during pregnancy that can result in a range of undesirable outcomes. From a psychological standpoint, one of the behavioral components of avoidance connected to birthing fear, according to Elisabet Rondung et al. (3), would be complete avoidance of pregnancy or postponing of pregnancy. It can be troublesome for countries and districts with low birth rates to halt the worse. Fear of childbirth (FOC) has also led to a surge of cesarean sections and requests that aren't medically necessary (4). Though cesarean is a surgical procedure for women who are having difficulty giving birth vaginally, it leads to undesirable consequences for both the newborn and the mother. Febrile condition, dysfunctional labor, and postpartum complications were all more common in women who had a cesarean section than in women who had a vaginal delivery (5). In terms of newborn growth, autistic spectrum disorders and childhood obesity were more likely in children born by cesarean section than in children born vaginally (6, 7).

Fear of childbirth is frequently characterized as a phenomenon within the anxiety domain (3). Cognitive components of anxiety are a typical part of established psychological models of anxiety of disorders (8). Negative attitudes and expectations about oneself, others, the world, or the future are among the cognitive components (3). Evidence have showed some links between Positive Affect (PA) and the cognitive components. Positive Affect is the degree to which a person feels energetic, active, and alert. A high degree of energy, total concentration, and enjoyable engagement are all characteristics of high PA (9). Positive emotions have an impact on how people view the world as well as how they think about it (10). As can be seen, PA might influence people's views toward the environment around them, as well as their cognitive components and their fear of childbirth.

Music is ubiquitous in everyday life, and it appears to have a significant impact on people's mental health. Historically, the term “musical activities” is related to activities proceeding with music. Singing, dancing, listening to music, and playing instruments are among musical activities (11). The belief that music practice and engagement can elicit beneficial emotional reactions is widely held (12). This was exemplified in a randomized control study, which discovered that frequent musical activities enhanced mood and cognition in dementia patients (13). Furthermore, a controlled study of pregnant women found that listening to music helps lower tension and anxiety (14). As a result, it's a legitimate assumption that musical activities will have an impact on positive affect and FOC.

In the previous research, several ways were sought to alleviate FOC such as exercise, self-efficacy, and art therapy (15–17). Few studies, on the other hand, have been undertaken on musical activities which consist of listening, dancing, singing, and playing instruments. Most importantly, it provides a more thorough understanding of music compared to single music listening. To our knowledge, it is quite possible that musical activities can explain at least some of the variability in FOC and open up pathways for early interventions.

Positive and negative affect have regularly emerged as two main and generally separate characteristics, which is interesting to observe. Positive affect is related to social activity and the frequency of pleasant events, while Negative Affect (NA) is not (9). Musical activities are thought to increase PA because they are pleasant events, although this has never been proven in previous research. As a result, measuring the impact of musical activities on PA is fairly valuable.

Little is known about the relationship between the musical activities and the PA to the FOC. A randomized and controlled trial revealed that music was effective for anxiety alleviation among pregnant women at high risk (18). A small qualitative study conducted among seven women suggested that creative activities during pregnancy, including dancing and singing, may boost their emotional and spiritual wellbeing (19). Examinations of the musical activities' effect on the PA during pregnancy are warranted. To date, there were few published studies of the relationships among musical activities, PA, and FOC.

Based on the evidence above, potential mediating effects may exist between the conceptual constructs. The Structural Equation Modeling (SEM) can provide several advantages in the area of mediation study, in addition to assessing the links among musical activities, the PA, and the FOC. It may, for example, easily interpret and estimate latent variables such as musical activities, PA, and FOC, which are difficult to observe directly (20). Hence, the purpose of this study was to investigate the associations of musical activities and PA with FOC and to explore the potential mediating effect of PA on the relationship between musical activities and FOC among pregnant women by applying a structural equation modeling approach.

## Materials and Methods

### Participants

A non-probability convenience sampling was used to identify prospective study pregnant women. We invited healthy pregnant women to participate in the study during a routine prenatal visit to one tertiary-grade A class hospital in Chongqing, China between March to August 2021. After the routine prenatal visit, all pregnant women collaborators were invited to participate in the online-based questionnaires through their mobile phones. A total of 1,139 questionnaires were collected. After eliminating outliers, the final sample was 1,053 according to the inclusion and exclusion criteria, which met the minimal sample requirement of 200 to construct a structural equation model (21). The process of eliminating outliers was presented in the statistical analysis part. Inclusion criteria: age >18 years old; singleton pregnancy; have certain cognitive comprehension ability and can complete the questionnaire independently. Exclusion criteria: previous diseases that change or affect physical activity, such as heart failure, respiratory system, kidney or liver disease, or neurological or musculoskeletal diseases that affect mobility; mental disorders that are difficult to cooperate with researchers; those explained to be unwilling to participate in this study or sign an informed consent form.

### Ethical Considerations

The study was approved by the Ethics Committee of the Third Affiliated Hospital of Chongqing Medical University (No. 202110). All study pregnant women were informed about the aim of the study and were assured that the information collected would be used only for research purposes and would remain anonymous. All women enrolled in this study provided informed consent before participation.

### Instruments

The questionnaire included four sections: demographic characteristics, a self-made music behavior questionnaire, the Positive affect subscale, and the Childbirth Attitudes Questionnaire (CAQ).

Demographic characteristics included women's age, height, pre-pregnancy weight, nationality, educational level, occupation, area of residence, monthly disposable income for pregnant women, family type, gestational age at the time of questionnaire completion, number of previous deliveries.

The self-made musical activities questionnaire was used to investigate the types of music activity (listening to music, playing musical instruments, singing, dancing), frequency (more than 5 times/week, 3–5 times/week, 1–2 times/week, never) and time (over 90 min, 61–90 min, 31–60 min, 10–30 min, under 10 min). The eight musical activities were rated on a scale of 0 = never to 3 = more than 5 times per week in terms of frequency, and on a scale of 0 = under 10 min to 4 = more than 90 min in terms of duration (11).

The CAQ, developed by Lowe (22) evaluates a pregnant woman's FOC. Wei Juan (23) tested the validity and reliability of the Chinese version of the questionnaire. The CAQ is a 16-item questionnaire, with a 4-point Likert scale. The item scores are summed to provide a total score (range: 16–64) with higher scores indicating higher levels of FOC, which is classified into four levels based on the total score: null (score 16–27), mild (28–39), moderate (40–51), and severe (52–64). The original CAQ has good reliability and validity.

The PA adopts a subscale of the positive and negative affect scale (PANAS) compiled by Watson (9) and revised by Huang Li (24). The subscale consists of 10 items and scores with five points, from “1” (completely inconsistent) to “5” (completely consistent). It consists of 10 adjectives that describe positive emotion. A high score of positive emotion indicates an energetic and happy emotional state, while a low score indicates indifference.

### Statistical Analysis

Item parceling is a method of merging single items and then using the resulting items as observed variables. This procedure was used in this study due to the non-normality of the data and the fitness of the SEM model. It is important to note that Principal Component Analysis (PCA) did not fit the data measuring the latent variable–musical activities. The data and proposed model, according to WU Yan (25), met the prerequisites of application for item parceling. The first step is to carry out the Confirmatory Factor Analysis (CFA). Then, the eight indicators of musical activities were ranked in descending order of loadings. Three adjacent-loading parcels were constructed by taking the sum of indicators that had contiguous loadings (26) (see loadings in Table 2). When the loadings appeared the cliff type change, the node for item parceling was selected. The indicators' loadings are correlation coefficients between latent variables and indicators.

All the statistical analysis was conducted by R version 4.0.5 (2021-03-31). Outliers and deviations from normality were also verified and excluded in continuous data, such as the initial weight before pregnancy and the stage of pregnancy. According to the field investigation, the data of height >2 meters and <1.5 meters were regarded as outliers. The normality of the quantitative variables was examined by Shapiro-Wilk normality tests. Descriptive statistics were used for the presentation of absolute and relative frequencies, mean (SD) or median (and quartiles). In case the data showed non-normality, Kruskal-Wallis tests were employed to determine whether or not there is a statistically significant difference between the medians of three or more independent groups, with further multiple comparisons using the *p*-value adjustment of the holm method. Wilcoxon rank-sum tests were used to compare two groups. Musical activities, positive affect, and fear of childbirth were latent variables in the SEM model and factors of the scales were the observed variables. The *lavaan* package (27) was used for the test of the associations of musical activities and positive attitudes with fear of childbirth and the test of the mediation effect.

## Results

The 1,053 pregnant women had a median age of 28 years (range 19–44 years) and a median gestation week of 28 weeks (range 1–40 weeks). The median PA score in all samples was 26 (range 10–43) and the median FOC score was 29 (range 16–64). The number of pregnant women with no fear of childbirth, mild fear of childbirth, moderate fear of childbirth, and severe fear of childbirth was 438 (41.60%), 415 (39.41%), 173 (16.43%), 27 (2.56%), respectively.

Table 1 presents an overview of PA scores/FOC scores in the pregnant women with different characteristics. The educational background distribution of 1,053 pregnant women was shown in Table 1. The statistical analysis of the PA scores based on the education showed that the PA and FOC scores were associated with the pregnant women's education (χ^2^ = 53.624, *P* < 0.01; χ^2^ = 10.265, *P* = 0.0164). Furthermore, Dunn method for multiple comparisons revealed that the pregnant women whose education were junior high school or below were measured significantly fewer PA scores than those who got a degree from postsecondary specialized college (*P* = 0.038) or bachelor degrees or higher (*P* < 0.01); the pregnant women whose education is high school (*P* < 0.01) or postsecondary specialized college (*P* < 0.01) got fewer PA scores than those who got bachelor degrees or higher. The pregnant women who got education from junior high school or below had lower FOC scores than those with Postsecondary Specialized College educational level (*P* = 0.040).

**Table 1:** PA scores and FOC scores by demographic characteristics.

**Characteristic**	**PA score/median** **(P25, P75)**	***N* (%)**	**statistics**	***P***	**FOC score/median** **(P25, P75)**	**statistics**	***P***
**Education**			53.624	<0.01		10.265	0.0164
Junior high school or below	24 (20, 28)	101 (9.59)			28 (21, 36)		
High school	25 (20, 28)	222 (21.08)			29 (23, 37)		
Postsecondary Specialized College	25.5 (21, 30)	360 (34.19)			31 (25, 39)		
Bachelor degree or higher	28 (24, 31)	370 (35.14)			28 (23, 36)		
**Working during pregnancy**				<0.01			0.706
No	25 (20, 29)	430 (40.84)			29 (24, 37)		
Yes	27 (22, 30)	623 (59.16)			29 (23, 36.5)		
**Parity**				0.377			<0.01
Parous women	26 (21, 30)	351 (33.33)			26 (20, 32)		
Nulliparous women	26 (21, 30)	702 (66.67)			31 (25, 38)		
**Discretionary income/month**			36.619	<0.01		0.592	0.898
≤RMB 3,000	25 (20, 29)	414 (39.32)			30 (24, 37)		
RMB 3,001–5,000	26 (22, 30)	468 (44.44)			29 (23, 36)		
RMB 5,001–10,000	28 (22, 31)	131 (12.44)			28 (23, 38)		
>RMB 10,000	30 (26.75, 34)	40 (3.80)			30.5 (22.25, 35)		
**Residence**			5.228	0.0732		0.296	0.862
City	26 (21, 30)	901 (85.57)			29 (23, 37)		
County and town	24 (20, 29)	137 (13.01)			30 (24, 36)		
Country	27 (21.5, 29.5)	15 (1.42)			29 (25.5, 36)		
**Family**				0.392			0.334
Unclear family	26 (21, 30)	538 (51.09)			30 (23, 38)		
Stem family	26 (22, 30)	515 (48.91)			29 (24, 35)		
**Gestational week**			5.504	0.0638		0.605	0.739
First trimester	25 (21, 29)	208 (19.75)			29.5 (23, 36)		
Second trimester	26 (21, 30)	264 (25.07)			29 (23, 26.25)		
Last trimester	26 (21, 30)	581 (55.18)			29 (24, 37)		

Positive affect scores were strongly associated with the monthly discretionary income of the pregnant mothers (χ^2^ = 36.619, *P* < 0.01). The monthly discretionary income distribution of 1,053 pregnant women is shown in Table 1. Dunn method for multiple comparisons revealed that PA scores in the 3,000 RMB group were lower than those in the 3,001–5,000 RMB (*P* < 0.01), 5,001–10,000 RMB (*P* < 0.01), and >10,000 RMB (*P* < 0.01) groups. The PA scores were lower in the 3,001–5,000 RMB group compared to the >10,000 RMB group (*P* < 0.01). The pregnant women with work during pregnancy had higher PA scores (*P* < 0.01). The nulliparous women had greater FOC scores than parous women (*P* < 0.01).

Three adjacent-loading parcels were constructed by taking the sum of indicators that had contiguous loadings (Table 2). When the loadings appeared the cliff type change, the node for item parceling was selected. So, the observational variables—Frequency of singing and Time of singing were parceled into M1; Frequency of listening to music and Time of listening to music were parceled into M2, and the others were parceled into M3.

**Table 2:** The observational variables' loadings of the musical activities.

	**Frequency of singing**	**Time of singing**	**Frequency of listening to music**	**Time of listening to music**
Musical activities	0.829	0.855	0.424	0.396
	Frequency of playing instruments	Time of playing instruments	Frequency of dancing	Time of dancing
Musical activtities	0.242	0.261	0.212	0.231

**Figure 1:**
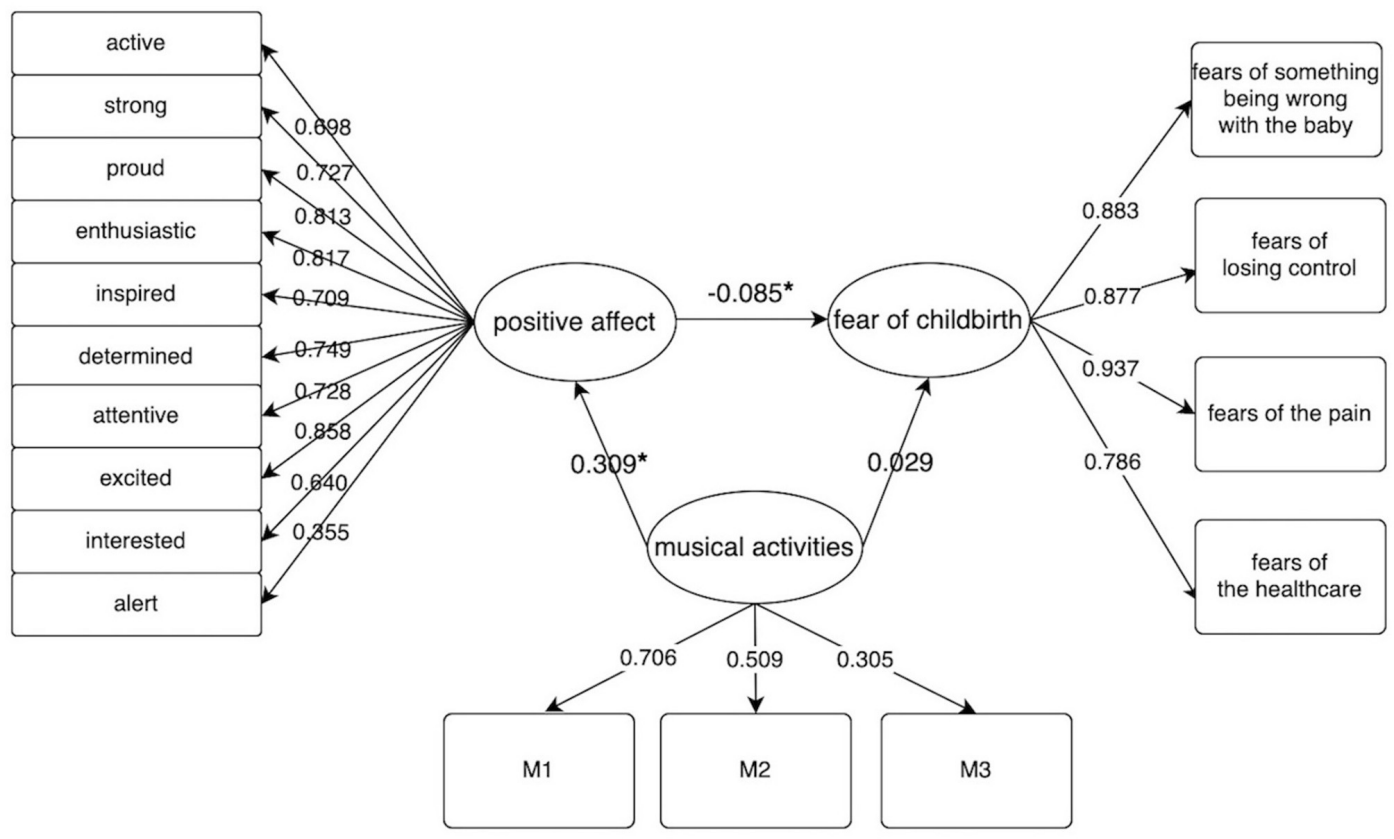
Overall structural model. Standardized path coefficients are presented. *stands for *P* < 0.05. SRMR (Standardized Root Mean Square Residual) = 0.048, RMSEA (Root Mean Square Error of Bayesian) = 0.063, CFI (Comparative Fit Index) = 0.950, TLI (Tucker-Lewis Index) = 0.941, chi-square/df ratio = 597.688/116=5.152.M1 = FOS (Frequency of singing) + TOS (Time of singing); M2 = FLM (Frequency of listening to music) + TLM (Time of listening to music); M3 = FPI (Frequency of playing instruments) + TPI (Time of playing instruments) + FOD (Frequency of dancing) + TOD (Time of dancing); Indirect effect: −0.026 (*P* = 0.030), total effect = 0.003 (*P* = 0.943).

Figure 1 presents the structural model with standardized coefficients (β), which indicates the associations of musical activities and PA with FOC. The final model showed a reasonably good model fit according to multiple SEM statistics and indices: the chi-square/df ratio was 5.152, the CFI was 0.950, the TLI was 0.941, the SRMR was 0.048, and the RMSEA was 0.063. The indices of CFI ≥0.95, TLI ≥0.95, and RMSEA ≤0.05 suggest a good fitting model according to empirical criteria (20).

Figure 1 showed that musical activities failed to directly affect FOC (β = 0.029, *P* = 0.514) while controlling for the mediator—PA. However, musical activities was positively associated with positive affect (β = 0.309, *P* < 0.01), which means more musical activities predict higher positive affect. Positive affect was negatively associated with FOC (β = −0.085, *P* = 0.019), which means higher positive affect predicts lower fear of childbirth. The indirect effect is statistically significant (β = −0.026, *P* = 0.030), which described the pathway from musical activities to FOC through PA. Interestingly, the path coefficient from the fear of childbirth to the fear of pain (β = 0.937, *P* < 0.01) is greatest from the measurable model of the fear of childbirth.

## Discussion

The goal of this study was to look at the links between musical activities and PA and FOC, as well as the potential mediating role of PA in the relationship between musical activities and FOC. The proposed construction and the median effect were both tested using structural equation modeling. This might the first study that we are aware of that reveals the mechanism linking musical activities to FOC through positive affect. The findings shed light on non-pharmaceutical and cost-effective interventions to alleviate childbirth fear. Soon, these methods are projected to be used as early interventions for pregnant women who are afraid of childbirth. In this study of 1,053 pregnant women in Chongqing, China, the prevalence of FOC was found to be 58.4%. Fear of childbirth and positive emotions were linked to some demographic variables.

### Demographic Characteristics, FOC and PA

Childbirth fear (CAQ >27) was reported by 58.4 percent of women in this study, with mild, moderate, and severe fear of childbirth rates of 39.41 percent, 16.43 percent, and 2.57 percent, respectively. The rate of extreme fear of childbirth is 2.57 percent, which is larger than the 0.7 percent reported in a previous study (28). According to a study conducted in Iran, 80.8 percent of nulliparous women are afraid of childbirth (29). In this study, the prevalence was lower than that. One possible explanation is that the study was limited to nulliparous women, who are more likely to be afraid than parous women (30). Different definitions, measures, and demographic data all contribute to the level of FOC, making comparisons between research tricky. However, we should point out that the majority of pregnant women suffer from FOC.

Parity and education were found to be connected with the fear of childbirth. There is some debate over whether these linked elements have an impact on FOC. Some research failed to detect significant connections between parity, education, and childbirth fear (28), whereas others discovered that education and parity were associated with women's fear of childbirth (29, 31). Our study confirmed that the educational level and parity were correlated with childbirth. The multiple comparison results showed that the pregnant women with only a junior high school degree or less had lower FOC scores than those with a Postsecondary Specialized College education. Women gain childbirth fear from a variety of sources, and women with higher education may have access to more knowledge from a variety of sources (32). Determining the utility of information, on the other hand, is never simple. As a result, ladies would be perplexed by the knowledge, and the abundance of information would only increase their worry. The relationship between parity and FOC could be explained by the fact that nulliparous women may be afraid of unfamiliar labor processes, as well as unanticipated pain and injury, while parous women may be afraid of childbirth due to previous traumatic labor experiences (33).

Being work during pregnancy, education, and discretionary income monthly were associated with positive emotions. Social relationships, fear for employment and social limitations influenced emotional state (34). Generally speaking, working and receiving higher education means more connections with people in society. That might explain, to the content, why women with work and higher educational levels achieved high PA scores. There is also a possibility that education was associated with work state in pregnancy because women with higher educational levels tended to achieve higher career goals in their life. From another perspective, being employed guarantees more discretionary income, which indicates a good preparation for the future. The result is consistent with the idea that overall preparedness evokes positive emotions (34). In sum, women should be encouraged to make more connections with people during pregnancy.

### Musical Activities, Positive Affect and Fear of Childbirth

On the question of expelling FOC, this study found the mechanism of how musical activities influence FOC through positive affect among pregnant women. Musical activities were related to the increase of positive affect, which was in turn related to the decrease of FOC. These results seem to be in line with other research which found music listening or music therapy reduced childbirth in pregnant women (17, 35). Musical activities have long been proven to increase positive affect as enjoyable events (9). When people listen to pleasurable music, neural pathways carry the signal from the ear to the inferior colliculi in the midbrain, and then to emotional centers in the cerebellum, nucleus accumbens (NAc), ventral tegmental area (VTA), and amygdala; these regions are activated, and dopamine levels are modulated (36). In this study, musical activities, rather than music therapy, seem to evoke positive emotions. This result provided further support for the early interventions that includes more aspects in music besides music listening. Moreover, positive emotions might reduce FOC. This result may be explained by the fact that PA would improve broad-minded coping, encouraging their novel ideas and actions (37). In another word, positive emotions make pregnant women do well with the forthcoming adversity—childbirth.

This might the first attempt to indicate the possible pathway that positive affect acts as a mediator between musical activities and FOC, even though there are some studies that revealed the relationship between music and FOC. We found no direct influence from musical activities to FOC. These results remind researchers of the importance of the effect of positive affect when people countered some obstacles. Positive affect triggers upward spirals reported by Fredrickson and Joiner (37) can partly support that opinion. To our knowledge, music therapy aims to make positive affect dominant, which allows your own emotions to cure mental problems. In previous studies, most of them focused on negative emotions such as the relationship between music and anxiety, depression, and FOC (38). However, in recent studies of the structure of affect, positive and negative affect have consistently emerged as two dominant and relatively independent dimensions (9), musical activities are supposed to influence positive affect not negative affect (9). What's more, positive affect might be more necessary to investigate for mental problems compared to negative affect. As a result, more interventions to evoke positive emotions are supposed to be discovered.

It is somewhat interesting that childbirth fear was most associated with pain fear. From a psychological perspective, it can be inferred that a cognitive belief—pain catastrophizing is correlated with childbirth (3). This finding has important implications for future interventions. For parous women, they might hold that belief because of their previous undesirable delivery experience; Positive affect might be beneficial for parous women to cure their undesirable experience. Nulliparous women may have catastrophized the pain due to a lack of understanding of the delivery procedure. Therefore, understanding the process would help them feel less scared.

In summary, the results suggested that musical activities would be an effective non-pharmaceutical intervention to enhance positive affect and further alleviate FOC among pregnant women. Probably, musical activities not only promote positive affect but also foster social connections or something else to expel FOC. There is no doubt about the fact that music has more potential than we can imagine as part of people's daily lives. There are also other reasons for us to pay more attention to this intervention. For instance, it is conveniently accessible to women and it is economically acceptable for nearly all pregnant women and so on.

The findings from this study have several implications for researchers interested in alleviating FOC among pregnant women. The results indicated that musical activities were positively related to positive affect, which was, in turn, negatively related to FOC. On the one hand, pregnant women are supposed to be encouraged to incorporate various musical activities into their daily lives. Pregnant women should take full advantage of the power of music because of its ease and economy. What's more, health education specialists might popularize the importance of musical activities. On the other hand, prevention programs may target pregnant women suffering from FOC. More specifically, the first step in prevention programs may be to arouse pregnant women's interest in music. Then, pregnant women should be encouraged to participate in multiple musical activities. This could improve positive affect so as to reduce FOC. In addition, fear of childbirth reduction programs may seek to combine musical activities triggering positive affect and other practices proved to reduce negative affect, which may amplify the individual effects. As also recommended above, future research could continue to explore more preventions to trigger positive affect.

These results need to be interpreted with caution due to some limitations. First of all, there can be other factors or structures such as women's personality, adverse experiences in childhood to reduce childbirth fear. These factors' effectiveness needs more research to be examined. Secondly, the sample was limited to one district, which implies the results should be generalized with caution. However, Chongqing, the study site, is a municipality directly under the Central Government of the People's Republic of China, a national central city, and a megacity. It is approved by The State Council as one of the important central cities in China, the economic center of the upper reaches of the Yangtze River, an important modern manufacturing base of the country, and a comprehensive transportation hub in southwest China (39). Finally, the cross-sectional study can not predict the causality between musical activities, positive affect, and fear of childbirth. Herein exists a possibility women with less childbirth fear tend to have more positive emotions and more musical activities. Thus, further longitudinal studies and causal inference studies are warranted.

Our results showed that relying solely on musical activities may not be adequate to change the FOC. In the present study, we offer a piece of evidence for future interventions. Despite the limitations, this might the first study that we are aware of that reveals the mechanism linking musical activities to FOC through positive affect. The findings reported here shed light on that musical activities could be an effective way to alleviate FOC through positive affect. This work also offers a valuable insight into the benefits and importance of positive affect. A natural progression of this work is to analyze the casual direction. Moreover, determining what kind of musical activities affect positive affect is needed.

## Data Availability Statement

The raw data supporting the conclusions of this article will be made available by the authors, without undue reservation.

## Ethics Statement

The studies involving human participants were reviewed and approved by the Ethics Committee of The Third Affiliated Hospital of Chongqing Medical University (No. 202110). Written informed consent was not provided because for the convenience of the survey, we informed each participant orally.

## Author Contributions

YF and XL developed the ideas, administrated the project and provided significant academic guidance on manuscript draft and interpretation, and revised the manuscript critically for intellectual content. SL researched the background literature, analyzed the data, and wrote the manuscript. YJ researched the background literature, investigated, and wrote part of the manuscript. HL, TZ, GZ, and LY administrated and supervised the project. All the authors approved the final version of the manuscript, ensured the accuracy and integrity of the work, and agreed to be accountable for all aspects of the work.

## Funding

This work was supported by the Natural Science Foundation General Project of Chongqing Science and Technology Bureau (No. cstc2020jcyj-msxmX0279) and the *Public Health Elite Program* funded by the School of Public Health and Management of Chongqing Medical University (GWJY2021018).

## Conflict of Interest

The authors declare that the research was conducted in the absence of any commercial or financial relationships that could be construed as a potential conflict of interest.

## Publisher's Note

All claims expressed in this article are solely those of the authors and do not necessarily represent those of their affiliated organizations, or those of the publisher, the editors and the reviewers. Any product that may be evaluated in this article, or claim that may be made by its manufacturer, is not guaranteed or endorsed by the publisher.
